# Evaluation of a natural S‐equol supplement in treating premenstrual symptoms and the effect of the gut microbiota: An open‐label pilot study

**DOI:** 10.1002/npr2.12234

**Published:** 2022-02-06

**Authors:** Takashi Takeda, Yasutaka Chiba

**Affiliations:** ^1^ Division of Women’s Health Research Institute of Traditional Asian Medicine Kindai University Osaka‐Sayama Japan; ^2^ 326473 Clinical Research Center Kindai University Hospital Osaka‐Sayama Japan

**Keywords:** dietary supplement, gut microbiota, isoflavone, premenstrual syndrome, women's health

## Abstract

**Aim:**

Premenstrual syndrome causes disturbances in many women's daily activities. Isoflavones might cause changes in the estrogen cycle by their selective estrogen receptor modulator‐like activities. Equol, which is a metabolite of a soy isoflavone, has greater biological activity than other soy isoflavones. In this preliminary study, we aimed to examine the effect of a natural S‐equol supplement (SE5‐OH) on premenstrual symptoms. The gut microbiota has recently been suggested to play an important role in brain function in psychiatric disease, such as depression. Therefore, we further aimed to evaluate the relationship of the effect of SE5‐OH and the gut microbiota at preintervention.

**Methods:**

Twenty women who showed premenstrual symptoms and were nonequol producers were enrolled in an open‐label, single‐arm, clinical study in which they received oral SE5‐OH for two period cycles. The Daily Record of Severity of Problems (DRSP) total score was evaluated during the intervention cycles. Before taking SE5‐OH, fecal samples were obtained and subjected to terminal restriction fragment length polymorphism analysis.

**Results:**

The response rate to treatment (≥50% reduction from baseline in the DRSP total score) was 10.5%. Post hoc analysis showed a significant improvement in the change in the DRSP total score (*P* = .008) and DRSP scores for four core premenstrual dysphoric disorder symptoms. Multiple regression analysis showed that the percentage improvement of the DRSP total score was positively related to *Bifidobacterium* and negatively related to *Clostridium cluster* IV.

**Conclusion:**

SE5‐OH supplementation may be an acceptable treatment for premenstrual symptoms. The intestinal microbiota may have an effect on SE5‐OH.

## Abstract 2

We examined the effect of a natural S‐equol supplement on premenstrual symptoms. Moreover, because research has also shown that the gut microbiota might play an important role in brain function, we further evaluated the relationship between the effectiveness of this S‐equol supplement and the pre‐intervention gut microbiota.

## Graphical abstract

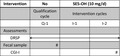

## 1 INTRODUCTION

Premenstrual syndrome (PMS) shows various mood, behavioral, and physical symptoms that are limited to the late luteal phase and disappear almost completely after the onset of menstruation.^1^, ^2^, ^3^ Epidemiological studies have shown a high prevalence of premenstrual symptoms (80%‐90%).^4^ Severe premenstrual symptoms interfere in personal or social relationships in approximately 5% of women.^5^ The Diagnostic and Statistical Manual of Mental Disorders (DSM‐5; American Psychiatric Association 2013) defined this severe type of PMS as premenstrual dysphoric disorder (PMDD).^6^ The precise pathophysiology of PMS remains unknown, but hormonal fluctuations, serotonergic dysfunction, impaired gamma‐aminobutyric acid function, a stressful life style, and bad eating habits have been suggested as possible causes.^7^, ^8^ For the treatment and management of PMS and PMDD, oral contraceptives (OCs) and serotonin reuptake inhibitors (SSRIs) are recommended and commonly prescribed.^3^, ^9^, ^10^ OCs and SSRIs are effective, but some physicians are reluctant to use them because of their adverse effects. In this situation, nonpharmacological approaches, such as changing dietary habits or supplements, might be recommended without serious adverse effects.^11^

Dietary habits of taking soy isoflavones and soy products can reduce the severity of menopausal symptoms and the risk of developing estrogen‐dependent diseases, such as breast cancer, osteoporosis, and cardiovascular disease.^12^, ^13^, ^14^, ^15^ Isoflavones have agonist–antagonist estrogen action and are similar to selective estrogen receptor modulators (SERMs).^16^ Equol is converted from daidzein by the metabolism of specific gut bacteria,^17^ and it is more biologically effective than other soy isoflavones. After eating soy, equol producers can produce equol from daidzein, and they are considered to benefit more from soy isoflavone consumption than nonequol producers.^18^ However, only 30%‐60% of people are equol producers.^19^ The equol‐producing ability is limited by the presence of specific gut microbiota that can produce equol from daidzein.

A recent report showed that an equol nonproducer is a significant risk factor for PMS.^20^ Equol supplementation for PMS could be a harmless treatment. With regard to alleviation of premenstrual symptoms, isoflavones might stabilize fluctuations in estrogen during the menstrual cycle by their SERM‐like activities.^21^ Therefore, isoflavones exert an estrogenic effect in the follicular phase and an antiestrogenic effect in the luteal phase. Some studies have also reported that isoflavones decrease progesterone concentrations during the luteal phase.^21^, ^22^, ^23^ A previous study reported that soy isoflavone supplementation was effective for specific premenstrual symptoms.^24^ A natural S‐equol supplement has been shown to be effective and safe for treating symptoms of menopause.^25^ However, no reports have described the efficacy of such a supplement for treating premenstrual symptoms. The gut microbiota has recently been suggested to play an important role in brain function.^26^ Therefore, in this study, we aimed to evaluate the relationship between the effect of an S‐equol supplement and the composition of the preintervention gut microbiota. Specifically, we examined the effect of a natural S‐equol supplement when used to treat premenstrual symptoms in nonequol producers.

## 2 METHODS

### 2.1 Institutional review board statement

We carried out the study in accordance with the principles defined in the Helsinki Declaration. The Ethics Committee of Kindai University approved the trial protocol (29‐018).

### 2.2 Trial design

This study was an open‐label, single‐arm, clinical study. This trial was registered with the University Hospital Medical Information Network (UMIN) centre (ID UMIN000033473).

### 2.3 Settings and participants

This trial was conducted at Kindai University Hospital. We advertized the trial in local free papers and posters that were displayed in Kindai University Hospital for recruiting participants. The study participants were recruited from June to September 2017. All of the participants received oral and written information about the study.

At enrollment, information on age, body weight, and height were collected. We also collected data on a previous history of allergies to food or medicine. The body mass index was calculated by dividing weight in kilograms by height in meters squared.

Eligible criteria for the trial were as follows: age of 20‐45 years; presence of premenstrual symptoms and fulfilment of the “moderate‐to‐severe PMS” or “PMDD” criteria evaluated by the Premenstrual Symptoms Questionnaire (PSQ)^27^; menstrual cycles are regular (25‐38 days); a soy challenge test determined a nonequol producer; without OC use for 4 weeks before entering study; and without coincidental therapy for PMS, including antidepressants, herbal medicines, or supplements, for 4 weeks before entering the study. The PSQ was developed to screen for premenstrual symptoms and has good reliability and validity.^27^, ^28^ The PSQ was established by translation of the DSM‐4 PMDD criteria into a severity rating scale in Japanese. Women were divided into three groups by their premenstrual symptoms according to the previously reported criteria of those with PMDD, moderate‐to‐severe PMS, or no/mild PMS.

Study participants were excluded for the following reasons: allergy to soybeans or soy products; presence of neuropsychiatric disorders; presence of severe PMS symptoms that interfered with work, usual activities, or relationships; and severe disease, such as hepatic failure, heart failure, kidney failure, or carcinogenic disease. Women with severe PMS were excluded from the study because they should be treated with medicine, such as an OC and SSRI, from an ethical perspective.

### 2.4 Interventions

The intervention was administration of a natural S‐equol supplement (SE5‐OH).^25^ During the intervention, the study participants received oral SE5‐OH in the form of two tablets twice daily from Day 5 (±2) of the menstruation cycle until the start of the next menstruation cycle, for two continuous cycles. Each SE5‐OH tablet contained 2.5 mg S‐equol, 0.04 mg daidzein, 0.04 mg genistein, 1.33 mg glycitein, 300 mg protein, 200 mg fat, and 375 mg carbohydrate. Otsuka Pharmaceutical Co., Ltd. (Tokyo, Japan) supplied these products for free. The participants were required to record daily whether they took these tablets.

### 2.5 Study procedure

**FIGURE 1:**
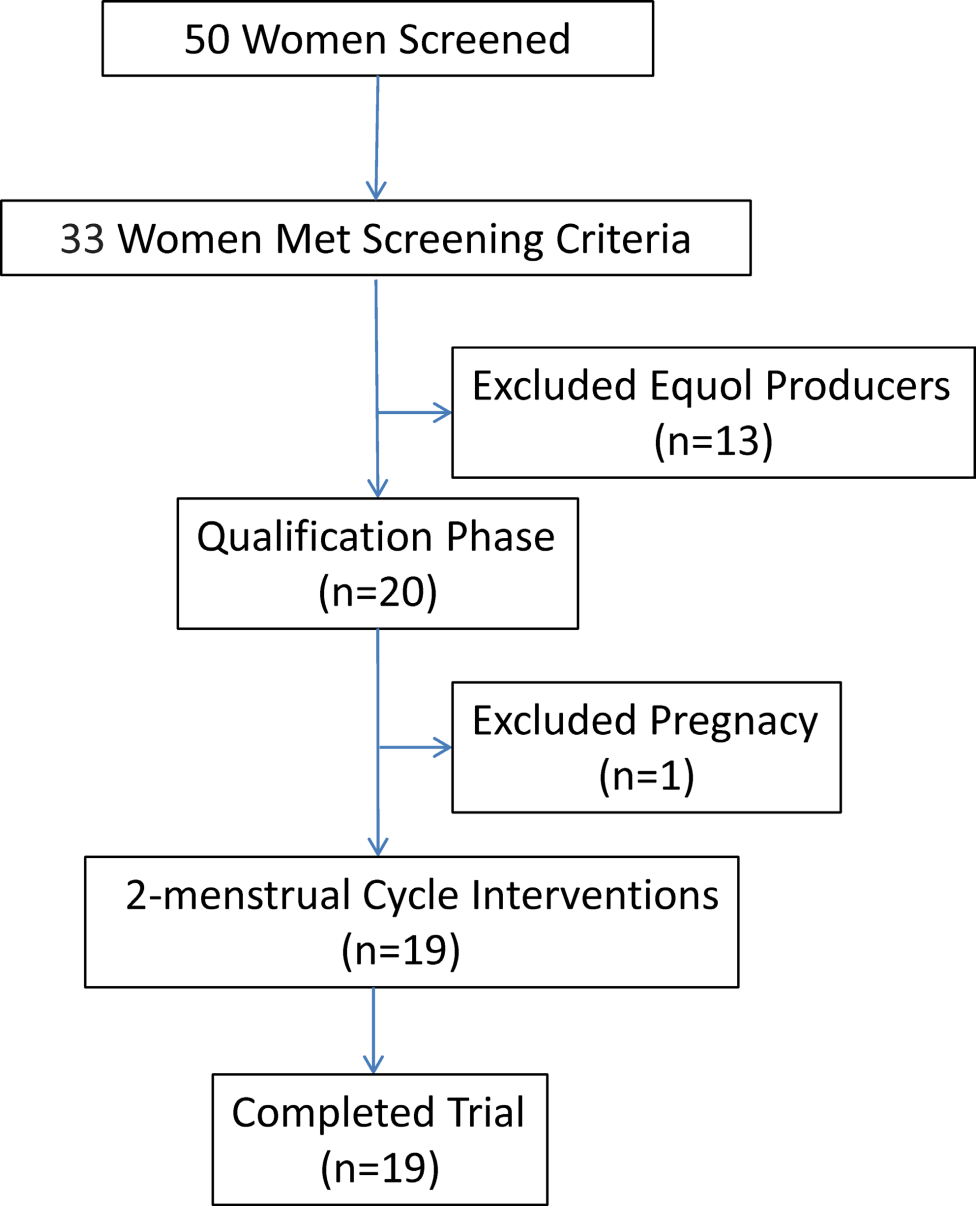
Study flow chart

A flow diagram of the study is shown in Figure 1. Fifty subjects were initially enrolled. Of these, 33 women met the screening criteria, and 13 equol producers were excluded. Among the 33 subjects who met the screening criteria, 20 entered the qualification phase. One woman was excluded during the qualification phase because she was pregnant. Ultimately, 19 women completed the trial.

**FIGURE 2:**
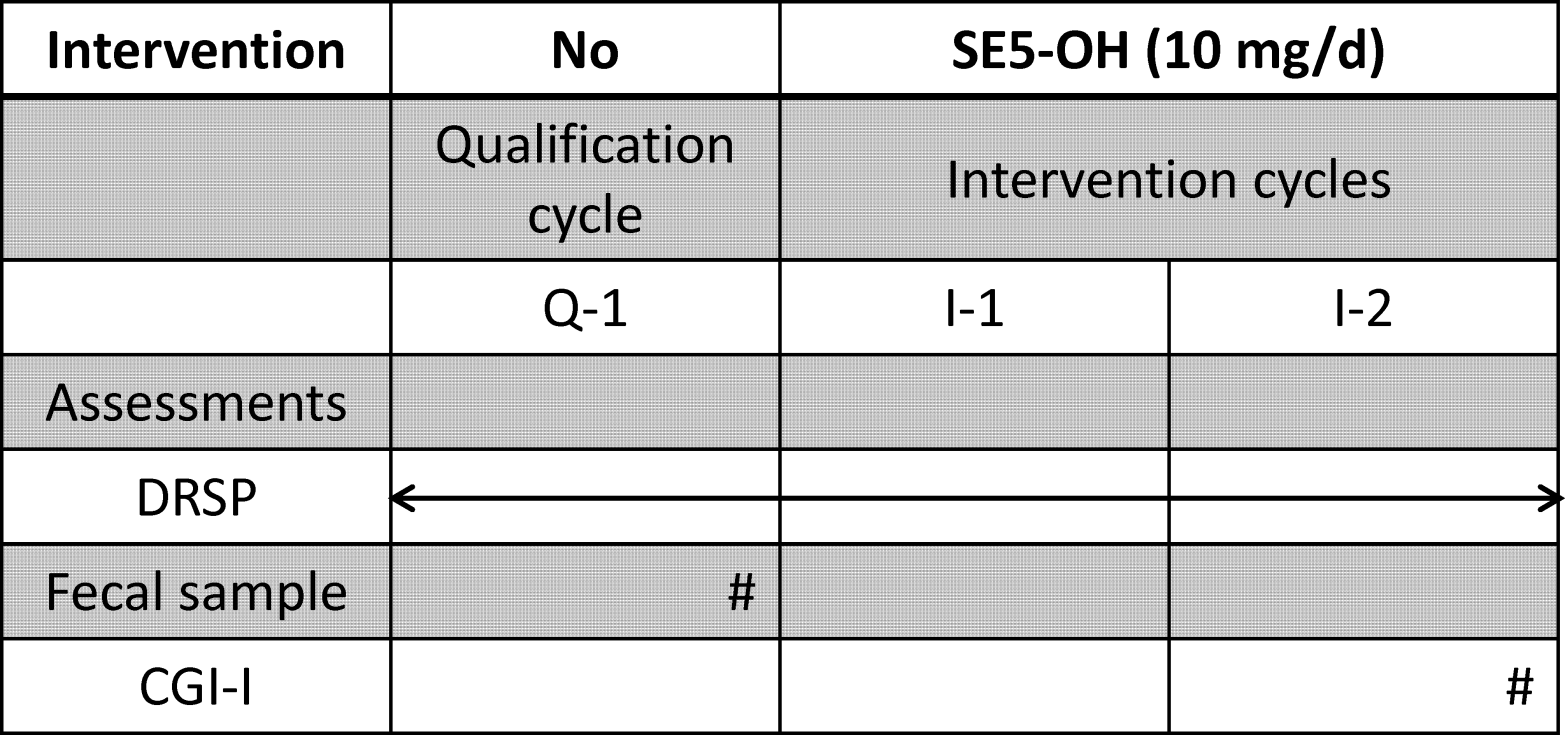
Schedule of enrolment, interventions, and assessments. CGI‐I, Clinical Global Impression–Improvement; DRSP, Daily Record of Severity of Problems; I‐1, first intervention cycle; I‐2, second intervention cycle; Q‐1, qualification cycle; SE5‐OH, natural S‐equol supplement

The time schedule of the study is shown in Figure 2. The study consisted of a qualification cycle (Q‐1) and two intervention cycles. We determined the equol production status of the subjects by a soy challenge test. The participants were instructed to eat a soy‐based food with approximately 50 mg of isoflavones for lunch and dinner and to collect the first morning urine samples in the next morning. In case of incidental bacterial infection, we scheduled the soy challenge test at least 1 week after antibiotic treatment to avoid an antibiotic effect on the gut microbiota. No women were pretreated with antibiotics in this trial. The equol concentration was measured by high‐performance liquid chromatography using a modified method described by Lundh et al.^29^ The detection limit of equol in this assay is 0.85 nmol/mL. The ratio of urine equol to daidzein was calculated and expressed as log_10_. Equol producers were defined as above −1.75 by this scale.^30^

We selected only nonequol producers for the study. These women were requested to check their premenstrual symptoms using the Daily Record of Severity of Problems (DRSP) for three consecutive periods. The DRSP comprises 21 items regarding emotional and physical premenstrual symptoms and 3 items regarding functional impairment of social and life activities, all of which are rated for their severity on a 6‐point scale (1, not at all; 6, extreme).^31^ Japanese translation of the DRSP (DRSP‐J) and the use of DRSP‐J for our clinical trial were permitted by Endicott et al,^31^ who designed this scale. The back‐translated version was verified for accuracy and equivalence by Endicott et al.^31^ The DRSP‐J was linguistically validated according to the general cross‐cultural adaptation process.^32^ Cronbach's alpha coefficient of the DRSP was 0.956 in this study.

Before the subjects began taking SE5‐OH, fecal samples were collected in sampling tubes containing guanidine thiocyanate solution (Techno Suruga Laboratory Co., Ltd., Shizuoka, Japan). Extracted bacterial DNA was subjected to terminal restriction fragment length polymorphism (T‐RFLP) analysis by Techno Suruga Laboratory Co., Ltd. T‐RFLP analysis is an established method for classifying gut microbiota based on 16S ribosomal RNA‐based methods^33^ and was performed as previously described.^34^, ^35^ Ten groups of gut microbiota components were classified as follows: *Bifidobacterium*, *Lactobacillales*, *Bacteroides*, *Prevotella*, *Clostridium* cluster IV, *Clostridium* subcluster XIVa, *Clostridium* cluster IX, *Clostridium* cluster XI, *Clostridium* cluster XVIII, and others. Each operational taxonomic unit was calculated as a percentage of the total operational taxonomic unit area and expressed as a percentage of the area under the curve.

### 2.6 Outcome measure

The primary outcome measure was the total score for the 21 symptoms evaluated by the DRSP. The average scores for each of the 21 items during the premenstrual phase (Days −5 to −1 before menstruation) were added together to generate the average premenstrual DRSP total score (21‐126 points). The primary efficacy variable was the difference between the DRSP total score from Q‐1 and the DRSP total score from the last intervention cycle (I‐2). The proportion of responders (defined as those with a ≥ 50% reduction from baseline in the DRSP total score) was then calculated. The secondary outcome measures were the Clinical Global Impression–Improvement (CGI‐I) scale score at the end of treatment and safety/adverse event assessments. The CGI‐I scale is a single‐item measure that consists of a 7‐point scale (from 1, very much improved, to 7, very much worse). We defined the response group as participants with a score of 1 (very much improved) or 2 (much improved).

Although we selected the participants who fulfilled the PSQ criteria for either moderate‐to‐severe PMS or for PMDD, the DRSP total score in Q‐1 was lower than expected. Six subjects had a DRSP total score of <42 in Q‐1, which made evaluating a ≥ 50% reduction from baseline in these subjects impossible. Therefore, we performed a post hoc analysis of the DRSP score. We evaluated the change in the DRSP total score and the DRSP score for each PMS symptom from Q‐1 to I‐2. We further calculated the percentage improvement (%improvement) of the DRSP total score. The %improvement of the DRSP total score was calculated as follows: (DRSP total score at Q‐1 − DRSP total score at I‐2) / DRSP total score at Q‐1.

### 2.7 Sample size calculation

No previous study has analyzed the effect of SE5‐OH on premenstrual symptoms. We hypothesized a threshold response rate of 20% and an expected response rate of 55%. Using these assumptions, the required sample size was calculated as 17, with a significance level of 2.5% (one‐sided) and a power of 80%, and Fisher's exact test was applied. Accounting for a few dropouts, the target sample size for subjects entering Q‐1 was set as 20.

### 2.8 Data analysis

All efficacy variables were analyzed for the full analysis set, which was defined as those who had a ≥ 1‐day DRSP total score measurement for Q‐1 and I‐2 without major aberration in the protocol. The safety analysis set comprised subjects who received the tablets at least once and who underwent the safety assessment.

Continuous variables are presented as means and standard deviations, and categorical variables are presented as percentages. The treatment response rate and CGI‐I score were analyzed with the statistical software SAS 9.4 (SAS Institute, Cary, NC, USA). The remaining data were analyzed with the statistical software JMP 13.0.0 (SAS Institute). Post hoc analysis of the DRSP score was performed by the Wilcoxon signed‐rank test. Multiple regression analysis was used to examine the effect of the gut microbiota on the %improvement in the DRSP total score. Variables that were predictive at a *P* value of <.20 were introduced into the step‐wise model.

## 3 RESULTS

The prevalence of equol producers was 38.2% (13/34 subjects). This rate was not significantly different from that of 41.8% (41/98), which has been reported previously among Japanese women^20^ (*P* = .437, as determined by Fisher's exact test).

Table 1 shows the characteristics of the study population. The mean age of the participants was 35.0 years (range, 20‐44 years).

**TABLE 1:** Baseline characteristics of the study population included in the intervention cycles

Characteristics	
Age, years	35.0 ± 7.8
Body weight, kg	52.2 ± 8.3
Height, cm	157.2 ± 4.9
BMI, kg/m^2^	21.0 ± 2.0
Allergies	0 (0.0)

Note

Data are presented as mean ± standard deviation or *n* (%).

Abbreviation: BMI, body mass index.

For the primary efficacy variable, the treatment response rate (≥50% reduction from baseline in the DRSP total score) was 10.5% (2/19) (95% confidence interval, 1.3%–33.1%). For the secondary efficacy variable, the clinical response analysis based on CGI‐I scores showed a 26.3% response rate (5/19) (95% confidence interval, 9.2%–51.2%).

**FIGURE 3:**
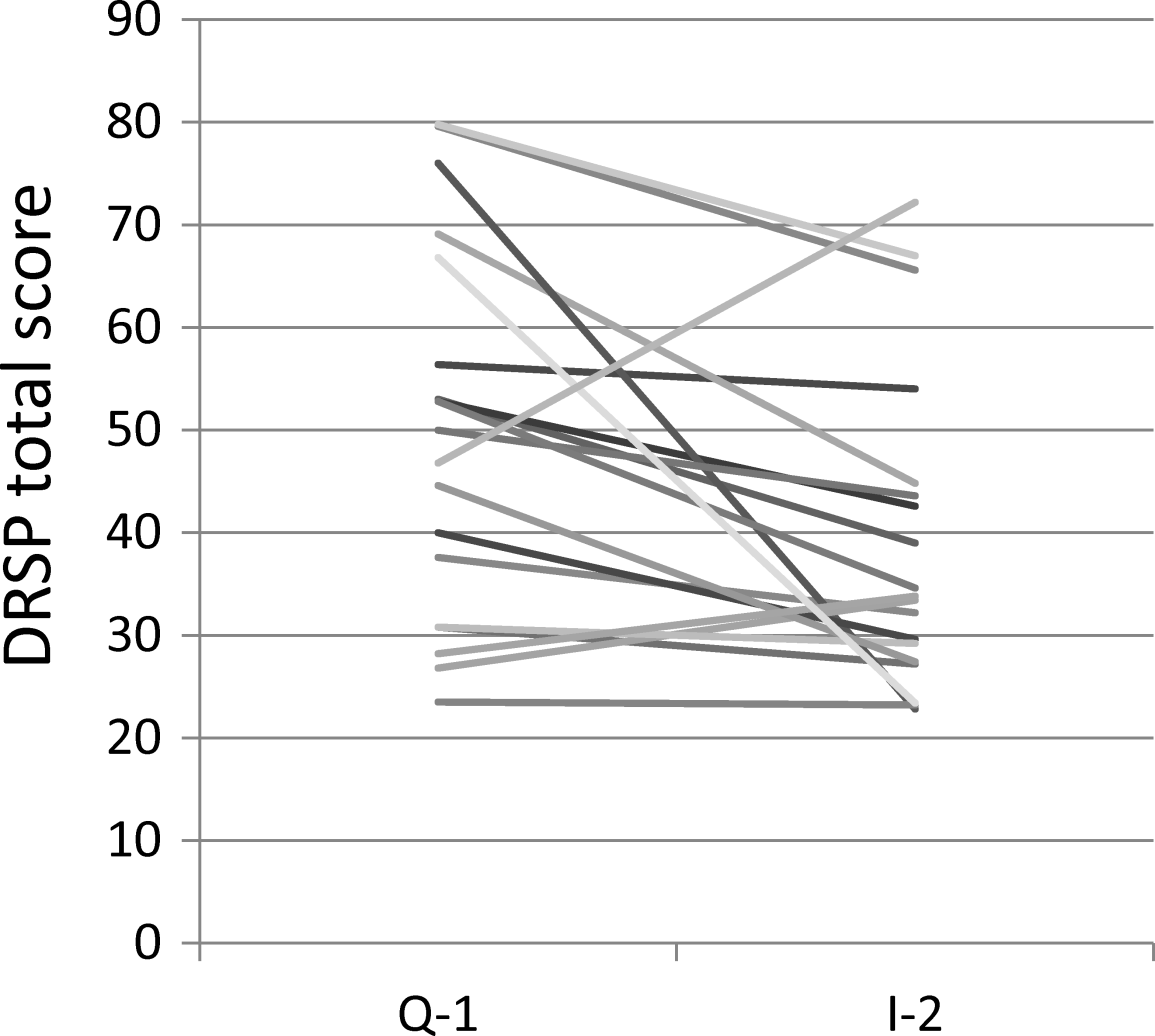
Changes in the DRSP total score before and after treatment with a natural. DRSP, Daily Record of Severity of Problems; I‐2, second intervention cycle; Q‐1, qualification cycle; S‐equol supplement

Post hoc analysis of the DRSP score was then performed. The changes in DRSP total scores from Q‐1 to I‐2 are shown in Figure 3. There was a significant reduction in the DRSP total score from Q‐1 to I‐2 (from 49.8 ± 18.1 to 39.2 ± 15.3; *P* = .008, as determined by the Wilcoxon signed‐rank test). The individual DRSP scores from Q‐1 to I‐2 were significantly lower after SE5‐OH treatment compared with baseline for each premenstrual symptom, except for “fatigue or lack of energy,” “overeating or food cravings,” and “insomnia or hypersomnia” (Table 2).

**TABLE 2:** Changes in the DRSP score for individual symptoms from baseline to the endpoint

Symptoms and distinct items	Q‐1	I‐2	Mean difference	95% CI	*P*
1. Depression	6.8	5.2	−1.6	−3.15 to −0.16	.031*
a. Felt depressed, sad, “down,” or “blue”	2.5	1.9	−0.6	−1.08 to −0.05	.036*
b. Felt hopeless	2.0	1.4	−0.6	−0.97 to −0.23	.001*
c. Felt worthless or guilty	2.4	1.9	−0.5	−120 to 0.23	.103
2. Anxiety Felt anxious, tense, “keyed up,” or “on edge”	2.6	2.0	−0.6	−1.17 to −0.09	.025*
3. Mood swings	5.4	4.1	−1.3	−2.50 to −0.11	.013*
a. Had mood swings (eg, suddenly felt sad or tearful)	2.7	2.0	−0.7	−1.40 to −0.09	.017*
b. Was more sensitive to rejection or my feelings were easily hurt	2.7	2.1	−0.6	−1.17 to 0.01	.024*
4. Irritability	5.2	4.0	−1.2	−2.41 to −0.04	.024*
a. Felt angry, irritable	3.0	2.4	−0.6	−1.33 to 0.14	.053
b. Had conflicts or problems with people	2.2	1.6	−0.6	−1.13 to −0.13	.012*
5. Decreased interest Had less interest in usual activities (eg, work, school, friends, and hobbies)	2.2	1.7	−0.5	−0.93 to −0.04	.014*
6. Difficulty concentrating Had difficulty concentrating	2.1	1.5	−0.6	−0.93 to −0.19	.005*
7. Fatigue or lack of energy Felt lethargic, tired, fatigued, or had a lack of energy	2.9	2.4	−0.5	−1.04 to 0.11	.087
8. Overeating or food cravings	4.2	3.9	−0.3	−157 to 1.06	.820
a. Had increased appetite or overate	2.1	2.1	−0.0	−0.68 to 0.68	1.000
b. Had cravings for specific foods	2.5	2.0	0.5	−1.13 to 0.31	.202
9. Insomnia or hypersomnia	5.3	4.3	−0.9	−1.91 to 0.01	.184
a. Slept more, took naps, and found it hard to get up when intended	2.8	2.3	−0.5	−0.90 to −0.02	.078
b. Had trouble getting to sleep or staying asleep	2.5	2.0	−0.5	−1.12 to 0.16	.202
10. Feeling overwhelmed	4.4	3.1	−1.3	−2.45 to −0.16	.017*
a. Felt overwhelmed or that I could not cope	2.1	1.5	−0.6	−1.17 to −0.10	.017*
b. Felt out of control	2.3	1.7	−0.7	−1.31 to −0.03	.038*
11. Physical symptoms	8.5	6.8	−1.7	−2.90 to −0.47	.009*
a. Had breast tenderness	2.0	1.7	−0.3	−0.75 to 0.09	.107
b. Had breast swelling, felt “bloated,” or had weight gain	2.4	2.1	−0.3	−0.80 to 0.25	.089
c. Had headache	2.1	1.6	−0.5	−0.91 to −0.14	.012*
d. Had joint or muscle pain	2.0	1.5	−0.6	−0.93 to −0.19	.006*

Abbreviations: CI, confidence interval; DRSP, Daily Record of Severity of Problems; I‐2, last intervention cycle; Q‐1, qualification cycle.

*P < .05.

All four core symptoms of PMDD (depression, anxiety, mood swings, and irritability) were significantly improved after SE5‐OH treatment compared with baseline (Table 2).

The composition of the fecal microbiota based on T‐RFLP analysis is shown in Table 3.

**TABLE 3:** Composition of the fecal microbiota based on terminal restriction fragment length polymorphism analysis

Bacteria	%
*Bifidobacterium*	16.9 ± 6.8
*Lactobacillales*	6.4 ± 6.9
*Bacteroides*	46.6 ± 11.2
*Prevotella*	0.2 ± 0.8
*Clostridium* cluster IV	4.4 ± 3.8
*Clostridium* subcluster XIVa	11.6 ± 4.3
*Clostridium* cluster IX	4.6 ± 4.3
*Clostridium* cluster XI	0.4 ± 0.9
*Clostridium* cluster XVIII	1.9 ± 1.9
Others	7.1 ± 3.4

Note

Data are presented as mean ± standard deviation.

The association between the preintervention gut microbiota and %improvement in the DRSP total score is shown in Table 4.

**TABLE 4:** Multiple regression analysis for calculating the weight of the microbiota toward percentage improvement in the DRSP score

	β	SE	*P*	Standardized β	VIF
*Bifidobacterium*	2.36	0.96	.0275	0.548	1.235
*Clostridium* cluster IV	−3.54	1.65	.0497	−0.433	1.012
*Clostridium* subcluster XIVa	2.65	1.42	.0822	0.416	1.225

Abbreviations: DRSP, Daily Record of Severity of Problems; SE, standard error; VIF, variance inflation factor; β, regression coefficient.

Multiple regression analysis showed that the %improvement in the DRSP total score was positively associated with *Bifidobacterium* and negatively associated with *Clostridium* cluster IV. Variance inflation factor analysis showed that multicollinearity was not present in this model.

No adverse events were reported during this study. SE5‐OH treatment was well tolerated.

## 4 DISCUSSION

To the best of our knowledge, this is the first report of the effect of SE5‐OH on premenstrual symptoms in Japanese women. Our previous study showed that the proportion of equol producers among patients with PMS (23.9%) was significantly lower than that in the control group (41.8%).^20^ In the present study, 38.2% of subjects were equol producers, which is comparable with the proportion of equol producers in the control group in our previous study. Our study was restricted to women with untreated PMS symptoms. Therefore, we speculate that the PMS symptoms experienced by our study group were not as severe as those experienced by patients with PMS receiving treatment at obstetrics and gynecology clinics. In fact, the degree of severity of PMS symptoms, as determined by the DRSP total score, was lower than expected in our study, and six women had a DRSP total score of <42 in Q‐1. Therefore, we were unable to evaluate a ≥ 50% reduction from baseline in these women.

Although we selected subjects who fulfilled the PSQ criteria for either moderate‐to‐severe PMS or for PMDD, the DRSP total scores in Q‐1 were lower than expected (range, 23.5‐79.8). Screening tools for PMS/PMDD, such as the PSQ, have a high sensitivity, but a low specificity.^36^ In the current study, we adjusted the screening process for our ongoing randomized, double‐blind, placebo‐controlled trial to more precisely evaluate premenstrual symptoms by using the DRSP for two consecutive menstrual cycles before inclusion.^37^

With regard to the alleviation of premenstrual symptoms, isoflavones might stabilize fluctuations in estrogen during the menstrual cycle and progesterone in the luteal phase,^21^, ^22^, ^23^ but the precise mechanism is not well known. Studies using ER‐β knockout mice have shown that ER‐β in the brain has anxiolytic effects.^38^ Studies in rats have also shown that the administration of equol has antidepressant effects.^39^ Equol selectively binds to ER‐β and has higher affinity for ER‐β than other isoflavones, such as daidzein and genistein.^40^ Equol may act directly on ER‐β in the brain to improve depression and anxiety symptoms of PMS.

Our study showed an association between the preintervention intestinal microbiota and the effect of SE5‐OH. We found that *Bifidobacterium* was positively associated with the effect of SE5‐OH *Bifidobacterium* has been suggested to have a beneficial effect on depressive disorders.^41^, ^42^ Although the precise mechanisms are not well understood, a previous report on a mouse model of depression showed that some *Bifidobacterium* strains increased levels of serotonin and its precursor 5‐hydroxytryptophan in the hippocampus.^43^ Therefore, *Bifidobacterium* may act in concert with the effects of SE5‐OH through neurotransmission. However, our study showed that *Clostridium* cluster IV was negatively associated with the effect of SE5‐OH *Clostridium* cluster IV is classified as a beneficial microbiota producing butyrate and plays an important role in gut homeostasis.^44^ A decrease in *Clostridium* cluster IV might play a major role in the pathogenesis of ulcerative colitis.^45^ There have been no reports on the relationship between *Clostridium* cluster IV and brain function. However, one report showed that the abundance of *Clostridium* cluster IV in the microbiota in patients with bipolar depression was higher than that in the microbiota in healthy controls.^46^ Further research is required to clarify the relationship of the intestinal microbiota and the efficacy of SE5‐OH.

Our study has several limitations. First, this study had an open‐label design without a placebo control group. On the basis of the results from this pilot study, we are currently studying the effect of SE5‐OH on premenstrual symptoms in nonequol producers in a randomized, placebo‐controlled, double‐blind study.^37^ Second, our study had a small sample size, which we believe was appropriate for a pilot study. In contrast, the target sample size for our ongoing study is 124 (62 in each group). Third, age‐related changes in ovarian function were not assessed by factors such as serum follicle‐stimulating hormone and anti‐Müllerian hormone. We cannot completely deny the possibility that women who had a relatively older age (ie, early 40s) had entered perimenopausal transition. One of the eligible criteria for this trial was a regular menstrual cycle (25‐38 days). Therefore, the participants were expected to have ovulatory menstruation. Women with ovulatory cycles can experience premenstrual symptoms, even though they are in perimenopausal transition.

## 5 CONCLUSION

This pilot study showed that SE5‐OH supplementation may be an acceptable treatment strategy for premenstrual symptoms. A randomized, double‐blind, placebo‐controlled trial based on the results from this study is currently underway,^20^ and is expected to generate definitive results.

## CONFLICT OF INTEREST

TT received lecture fees from Otsuka Pharmaceutical Co., Ltd., which is the manufacturer of SE5‐OH. Otsuka Pharmaceutical Co., Ltd. had no role in the study conception, study planning, data analysis, or data interpretation.

## AUTHOR CONTRIBUTIONS

Conceptualization, TT; Methodology, TT and YC; Software, TT and YC; Validation, TT and YC; Formal Analysis, YC; Investigation, TT; Resources, TT; Data Curation, TT; Writing–Original Draft Preparation, TT; Writing–Review and Editing, YC; Visualization, TT; Supervision, TT and YC; Project Administration, TT; Funding Acquisition, TT.

## APPROVAL OF THE RESEARCH PROTOCOL BY AN INSTITUTIONAL REVIEWER BOARD

The study was carried out in accordance with the principles outlined in the Helsinki Declaration. The Ethics Committee of Kindai University approved the trial protocol (29‐018).

## INFORMED CONSENT

Informed consent was obtained from all subjects involved in the study.

## INSTITUTIONAL REVIEW BOARD STATEMENT

The study was carried out in accordance with the principles outlined in the Helsinki Declaration. The Ethics Committee of Kindai University approved the trial protocol (29‐018).

## REGISTRY AND THE REGISTRATION NO. OF THE STUDY/TRIAL

UMIN000033473

## Data Availability

The raw dataset supporting these findings cannot be released to the public because it may contain confidential patient information. In accordance with Japanese ethical guidelines, the Ethics Committee of Kindai University has restricted the release of the raw dataset for this study. For data access requests, please contact the corresponding author.
